# In situ proximity ligation assay for analysing spatial interactions between ciliary proteins

**DOI:** 10.1186/s12860-025-00555-7

**Published:** 2025-10-16

**Authors:** Thorsten Pfirrmann, Ulrich Rüther, Christoph Gerhardt

**Affiliations:** 1 Institute of Molecular Medicine, Department of Medicine, Health and Medical University Potsdam, 14471 Potsdam, Germany; 2 Institute for Animal Developmental and Molecular Biology, Heinrich-Heine University Düsseldorf, 40225 Düsseldorf, Germany

**Keywords:** Cilia, Transition zone, Ciliopathy, Protein interaction, In situ proximity ligation assay, Fluorescence microscopy, Rolling circle amplification, Subcellular localization, Rpgrip1l

## Abstract

**Background:**

Cilia are small, hair-like structures on the surface of most eukaryotic cells. They are composed of distinct substructures: the basal body, the transition zone, and the axoneme. Proper ciliary function is crucial for human health, and defects can result in a group of disorders known as ciliopathies. Many ciliopathy-associated mutations affect genes encoding proteins of the ciliary transition zone, a key structural and regulatory region at the base of the cilium. Understanding the molecular composition and interactions within subciliary compartments, such as the transition zone, is essential to elucidate their role in ciliary function and disease.

**Results:**

Protein interaction studies have played a central role in uncovering the functional landscape of subciliary compartments. In this context, the in situ proximity ligation assay (in situ PLA) has emerged as a valuable tool to investigate whether two proteins are located in close proximity (less than 40 nm) within the cellular environment, implying potential interaction. In situ PLA uses primary antibodies to recognise target proteins, followed by secondary antibodies conjugated with oligonucleotides (PLA probes). When two probes are sufficiently close, added circle-forming oligonucleotides can hybridise and ligate to form a circular DNA strand. This DNA circle serves as a template for rolling circle amplification, which is then detected through hybridisation with fluorescently labelled oligonucleotides. The resulting signals can be visualised using fluorescence microscopy, enabling precise spatial mapping of protein proximities in cells.

**Conclusions:**

The in situ PLA technique offers a powerful means of detecting protein proximities in subciliary compartments with high spatial resolution. This method supports the identification of novel protein interactions and contributes to a deeper understanding of subciliary architecture and its disruption in ciliopathies.

## Background

**Fig. 1:**
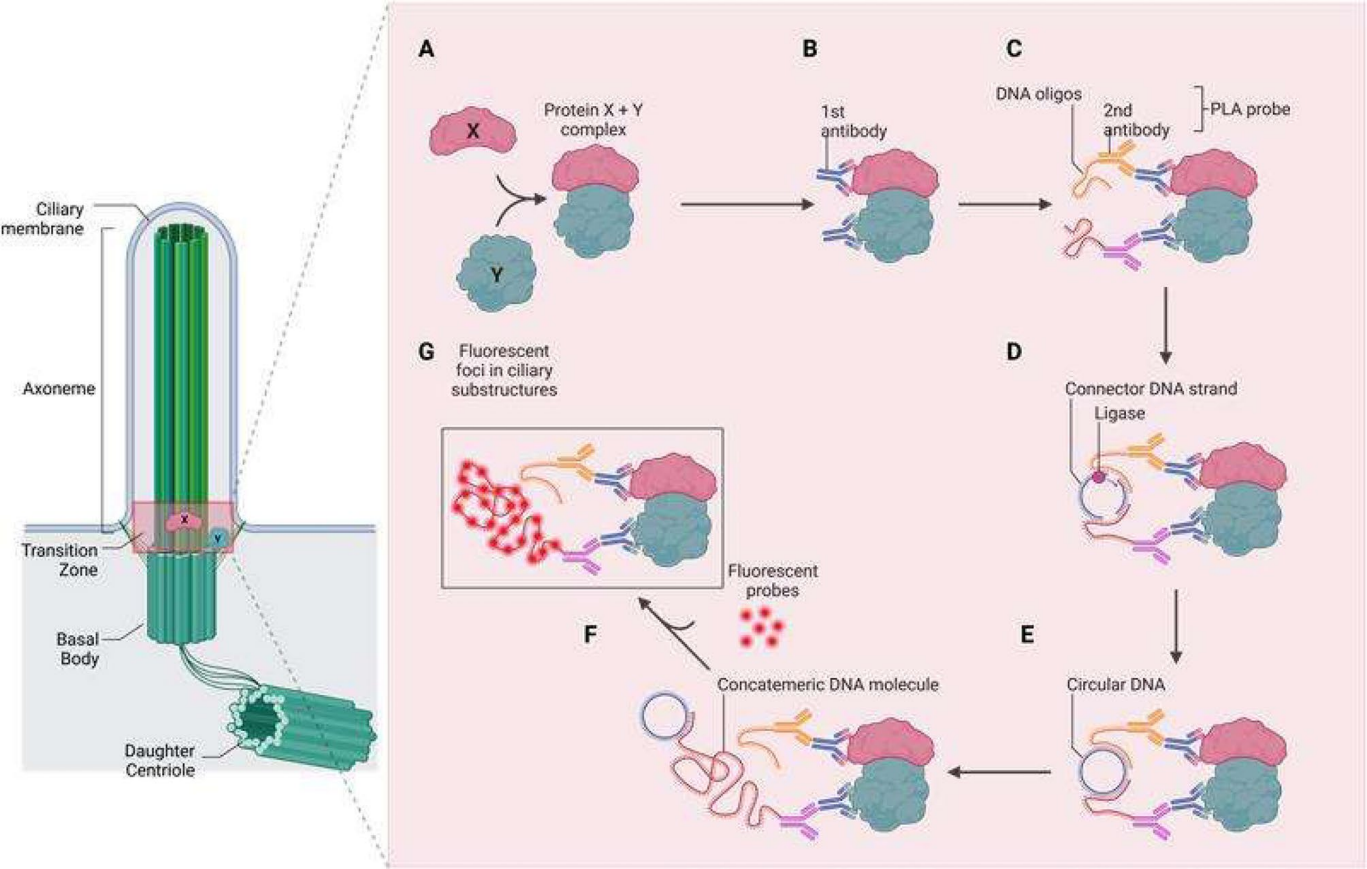
Schematic illustration of a primary cilium and an in situ PLA. A scheme of the primary cilium is shown on the left side of the figure. It consists of a microtubule scaffold referred to as axoneme (illustrated in green) which is anchored in the cell membrane by the basal body, the modified mother centriole near to the daughter centriole. At the proximal end of the axoneme is the transition zone (region marked in red) that acts as the ciliary gatekeeper regulating ciliary protein entry and exit and hence ciliary protein composition. The two proteins of interest are labelled with the letters X and Y and localise to the transition zone. The primary cilium is surrounded by the ciliary membrane. (**A**) Two proteins are in close proximity within the ciliary transition zone. (**B**) Incubation of the sample with primary antibodies which specifically bind to the target proteins. The primary antibodies have to be used from two different species. (**C**) Addition of PLA probes, each with a unique short DNA strand attached to it. (**D**) Hybridisation of two circle-forming DNA oligonucleotides and ligation to form a complete DNA circle. (**E**) Rolling circle amplification. (**F**) Following the amplification process, fluorescent oligonucleotides (red balls) bind to the amplified DNA to detect proteins being in close proximity. The images of Fig. 1 were created in BioRender. Pfirrmann, T. (2025) https://BioRender.com/z5m0ys2

Cellular processes are the elixir vitae of every organism, whether it is a plant, a fungus or an animal (including humans). They are precisely regulated by a coordinated expression pattern of numerous genes. These genes encode proteins which are part of signalling cascades and hence are deeply involved in the control of cellular processes e.g. by binding to the DNA (transcription factor function) or by binding to other proteins (protein-protein-interactions). The mediation of these signalling pathways in nearly all vertebrate cells is realised by a specialised cell organelle – the primary cilium. The primary cilium is a tiny cytoplasmic protrusion that appears on nearly all cells in vertebrates. Remarkably, the general public is more familiar with the function of motile cilia than with the function of primary (non-motile) cilia since motile cilia execute generally known transport processes, as for example the transport of mucus in the trachea or the movement of sperm. The dysfunction of both cilia subtypes results in severe human diseases referred to as ciliopathies. However, the number of ciliopathies caused by defective primary cilia is higher than the number caused by impaired motile cilia [1]. Generally, primary cilia can be structurally divided into three parts: the basal body, the transition zone and the axoneme (Fig. 1). The basal body is a modified centrosome from which the ciliary microtubule-based framework (axoneme) grows out. The axoneme consisting of nine doublet microtubules arranged in a circle gives stability to the cilium and conduces to the transport of proteins that are conveyed through the cilium. The intermediate range from the basal body to the axoneme is a short region of approximately 0.5 μm called transition zone. Many ciliopathies can be traced back to mutations of genes encoding transition zone proteins [1–4] arousing great interest in the assembly and the function of the transition zone. The identification of protein interactions between several transition zone proteins made a huge contribution to the current understanding of transition zone assembly and function [2, 5] demonstrating the meaning of protein-protein interaction studies in the ciliary research field.

Proximal labelling techniques, such as BioID, TurboID and APEX, have been used to map broad, dynamic ciliary protein interaction landscapes [6–8]. The BioID technique is based on the fusion of a ciliary protein to a biotinylating enzyme (originally BirA*, a mutated Escherichia coli biotin ligase). After the expression of this fusion protein in ciliated cells and after adding biotin to these cells, the ligase biotinylates lysine residues on proteins nearby to the fusion protein (labelling time: hours). Finally, the biotinylated proteins are captured and identified by mass spectrometry. The TurboID method represents an improvement on the BioID technique. It uses an engineered biotin ligase working with higher activity and hence enabling rapid labelling (labelling time: minutes). While BioID and TurboID require the action of a biotin ligase, the APEX technique functions by utilising an ascorbate peroxidase. A fusion protein consisting of a ciliary protein and an ascorbate peroxidase is expressed in ciliated cells, biotin-phenol is added and the cells are briefly exposed to H_2_O_2_. The APEX method generates highly reactive radicals which biotinylate proteins nearby the fusion proteins in a very fast way (labelling time: seconds). In the end, the biotinylated proteins are captured and analysed by mass spectrometry. BioID, TurboID and APEX are ideally suited proximity labelling techniques to identify novel interactions e.g. between ciliary proteins, but these methods do not provide any spatial information about these interactions.

In the year 2006, Söderberg and his colleagues developed a method called in situ proximity ligation assay in situ PLA) [9]. In contrast to the above mentioned proximal labelling techniques, the in situ PLA method adds spatial information about ciliary proteins localising in close proximity as it allows the detection of protein-protein proximity with high spatial resolution in fixed cells or tissues. If the two analysed proteins are located closer than 40 nm, it is visualised by a fluorescent signal. In situ PLA is performed on the basis of the endogenous proteins making in situ PLA to an ideally suited method for clinical studies. This is a major advantage of in situ PLA over methods that are dependent on labelled fusion proteins, such as for example the Förster resonance energy transfer (fluorescence resonance energy transfer, FRET) [10]. Additionally, in situ PLA is an excellent complement to interaction partner studies, such as yeast two-hybrid (Y2H) screen, co-immunoprecipitation (Co-IP) or tandem affinity purification (TAP). In contrast to these methods, in situ PLA enables the users to detect the subcellular location of the protein interaction.

To illustrate the mechanism of in situ PLA, a schematic overview is presented in Fig. 1: The two proteins of interest are recognised by specific primary antibodies (Fig. 1A, B). These antibodies can be derived from any species, provided that they are specific to the proteins of interest. Afterwards, the PLA probes are added to the protein-antibody complexes (Fig. 1C). These probes consist of secondary antibodies that are species-specific and conjugated to short, unique DNA strands. Importantly, the DNA-conjugated secondary antibodies allow researchers to employ standard primary antibodies, enabling the method’s broad applicability across different experimental systems. If the two proteins are in close proximity – typically within 40 nm – the DNA strands attached to the PLA probes interact upon the addition of two specially designed connector oligonucleotides (Fig. 1D). These connector oligonucleotides hybridise to the DNA strands on the PLA probes, and a ligation reaction is carried out to join the DNA ends, forming a circular DNA molecule. Subsequently, a rolling circle amplification (RCA) reaction is initiated from the ligated DNA circle (Fig. 1E). This amplification step generates a long single-stranded DNA product that remains tethered to the site of the original protein-protein interaction. The final step involves hybridisation of fluorescently labeled complementary oligonucleotide probes to the amplified DNA (Fig. 1F), enabling visualisation as discrete fluorescent dots under a fluorescence microscope (Fig. 1G).

This work aims to provide a detailed protocol for investigating protein proximities within or at cilia using in situ PLA.

## Methods

### Ethics statement

All animal procedures were performed in accordance with the relevant national guidelines for the Care and Use of Laboratory Animals (LANUV) and with approval from the authority for animal work at Heinrich Heine University Düsseldorf, Germany (Permit Number: O18/99).

### Animal care

The origin of the mouse breeding was acquired from Charles River Laboratories, Sulzfeld, Germany. All mice (*Mus musculus*) used in this study were maintained in the C3H background and housed at 22–24 °C on a 12/12 h dark-light cycle with food and water *ad libitum*. Pregnant mice were killed by qualified personnel using cervical dislocation.

### Cell culture

Mouse embryonic fibroblasts were isolated from single mouse embryos at embryonic day (E)12.5 using standard procedures [11]. Mouse embryonic fibroblasts (MEFs) were grown in DMEM supplemented with 10% foetal calf serum (FCS), 1/100 (v/v) L-glutamine (Gibco), 1/100 (v/v) sodium pyruvate (Gibco), 1/100 (v/v) non‐essential amino acids (Gibco) and 1/100 (v/v) pen/strep (Gibco) at 37 °C and 5% CO_2_. For induction of ciliogenesis, MEFs were grown to confluency and serum‐starved with medium containing 0.5% FCS for at least 24 h. DNA transfection was carried out by using Lipofectamine 2000 (Invitrogen) and by following the manufacturers’ guidelines.

### Blocking

### In situ PLA

The following equipment was used: pipettes (for transfer volumes ranging from 1 µl to 1000 µl), a laboratory shaker, an incubator set at + 37 °C, a suction apparatus, a moist chamber, freeze blocks for enzyme transport, a grease pencil for delimiting the reaction area (e.g. ImmEdge™ Pen, Vector Laboratories), staining jars, cover slips suitable for fluorescence microscopy, and a fluorescence microscope equipped with excitation/emission filters, a digital camera and image acquisition software.

Reagents were prepared using high-purity water (sterile filtered, e.g. MilliQ^®^). All solutions were prepared fresh or stored according to manufacturer specifications. Duolink^®^ In Situ Detection Reagents (Sigma-Aldrich, Munich, Germany) were used, in this study specifically the Detection Reagents Orange (excitation: 554 nm; emission: 579 nm). Other available variants include Detection Reagents Green (Excitation: 495 nm, Emission: 527 nm), Red (Excitation: 594 nm, Emission: 624 nm), and Far Red (Excitation: 644 nm, Emission: 669 nm). The kit components included ligation and amplification stocks (each 5×), ligase (1 U/µl), and polymerase (10 U/µl). Wash Buffers A and B were reconstituted in high-purity water and stored at room temperature or + 4 °C, as appropriate. Phosphate-buffered saline (PBS) was prepared from 4 mM NaH₂PO₄·H₂O and 16 mM Na₂HPO₄·2 H₂O (AppliChem, Darmstadt, Germany), and 150 mM NaCl (Fisher Scientific, Schwerte, Germany), pH-adjusted to 7.3 and autoclaved (121 °C, 2 bar, 30 min). Mowiol (Polysciences, Eppelheim, Germany) was used as a mounting medium and prepared by dissolving 5 g Mowiol in 20 ml PBS overnight with stirring, followed by the addition of 10 ml glycerol and centrifugation at 3,000 g for 15 min. A DAPI stock solution (Merck, Darmstadt, Germany) was used at 1 µg/ml final concentration. Triton X-100 (Sigma-Aldrich) was used for permeabilisation. The blocking solution consisted of 10% normal donkey serum in PBS with 0.1% Triton X-100.

In this work, in situ PLA was performed in MEFs transiently transfected with an IFT88-EYFP construct, a gift kindly provided by D.R. Beier (Center for Developmental Biology and Regenerative Medicine, University of Washington School of Medicine, Seattle, WA). This construct encodes an IFT88-EYFP fusion protein, present in the pJAG368 vector and expressed under the control of the SV40 promoter. Before the experiment is started, each sample has to be applied to a glass slide. The fixation of the cells samples depends on the conditions which the used primary antibodies prefer. In each of the following protocol steps, the samples have to be completely covered with the respective solution. Aspiration and removal steps are always performed with the help of a suction apparatus.

### Blocking

Samples were washed three times in PBS (1 min per wash), permeabilised with PBS containing 0.5% Triton X-100 for 10 min at room temperature, and again washed three times in PBS. Blocking was performed for at least 10 min at room temperature using 10% donkey serum in PBS containing 0.1% Triton X-100. During all steps, samples remained fully covered by solution and were protected from drying.

### Primary antibodies

Primary antibodies were diluted in a custom diluent suitable for the specific antibody pair. After removing the blocking solution, the antibody mixture was immediately added. Samples were incubated overnight at + 4 °C in a sealed moist chamber to ensure optimal antibody binding.

### PLA probes

On the following day, a moist chamber was pre-heated to + 37 °C. PLA probes were diluted 1:5 in Duolink Antibody Diluent or in the same buffer used for primary antibodies and pre-incubated at room temperature for 20 min. Importantly, PLA probes have to match the host of the primary antibodies. Samples were washed three times in PBS (10 min each on a shaker), followed by application of the PLA probe solution. Incubation was performed for 1 h at + 37 °C in the moist chamber.

### Ligation

The ligation solution was prepared by diluting the ligation stock 1:5 in high-purity water. Ligase was added at a 1:40 dilution immediately prior to use. After two washes in 1× Wash Buffer A (5 min each under gentle shaking), the ligation solution was applied to the samples and incubated at + 37 °C for 30 min.

### Amplification

The amplification stock was diluted 1:5 in high-purity water under low-light conditions. Polymerase was added at a 1:80 dilution. Samples were washed twice with Wash Buffer A (2 min each), and the amplification mix was applied. This amplification mix already contains the polymerase as well as the fluorescent oligonucleotides. Subsequently after the rolling circle amplification of the DNA, the fluorescent oligonucleotides bind to the newly formed DNA. Incubation was carried out for 100 min at + 37 °C in the dark to protect the fluorophores from photobleaching.

### Final washing steps

Following amplification, samples were washed twice with 1× Wash Buffer B (10 min each) and once with 0.01× Wash Buffer B (1 min), all at room temperature and in the dark. After removing the final wash buffer, samples were air-dried at room temperature in the dark.

### Mounting procedure

Coverslips were mounted using Mowiol containing 1 µg/ml DAPI, and slides were left to dry overnight in the dark. Air bubbles were carefully avoided during mounting.

### Image acquisition

Next day, the acquisition of single plane images and the data analysis were carried out at room temperature using a Zeiss Imager.A2 microscope, 100×, NA 1.46 oil immersion objective lens (Carl Zeiss AG), a monochrome charge-coupled device camera (AxioCam MRm, Carl Zeiss AG), and the AxioVision Rel. 4.8 software package (Carl Zeiss AG). The PLA signal was observed as distinct fluorescent punctae, indicating close proximity (< 40 nm) between the targeted proteins.

### Storage

Samples were stored at + 4 °C in the dark until analysis.

### Quantifications

No formal statistical hypothesis testing or power analysis was necessary, as the protocol was developed and optimised for qualitative and spatial assessment of protein proximities. Quantitative image analysis (e.g. PLA signal count per cell or per cilium) may be performed using standard image analysis software if required for comparative studies.

#### Figure preparation

Figure 1 was created by using BioRender.com. A BioRender Academic Publication License is available upon request. The preparation of all other figures was performed by using Adobe Photoshop and Microsoft PowerPoint.

## Results

**Fig. 2:**
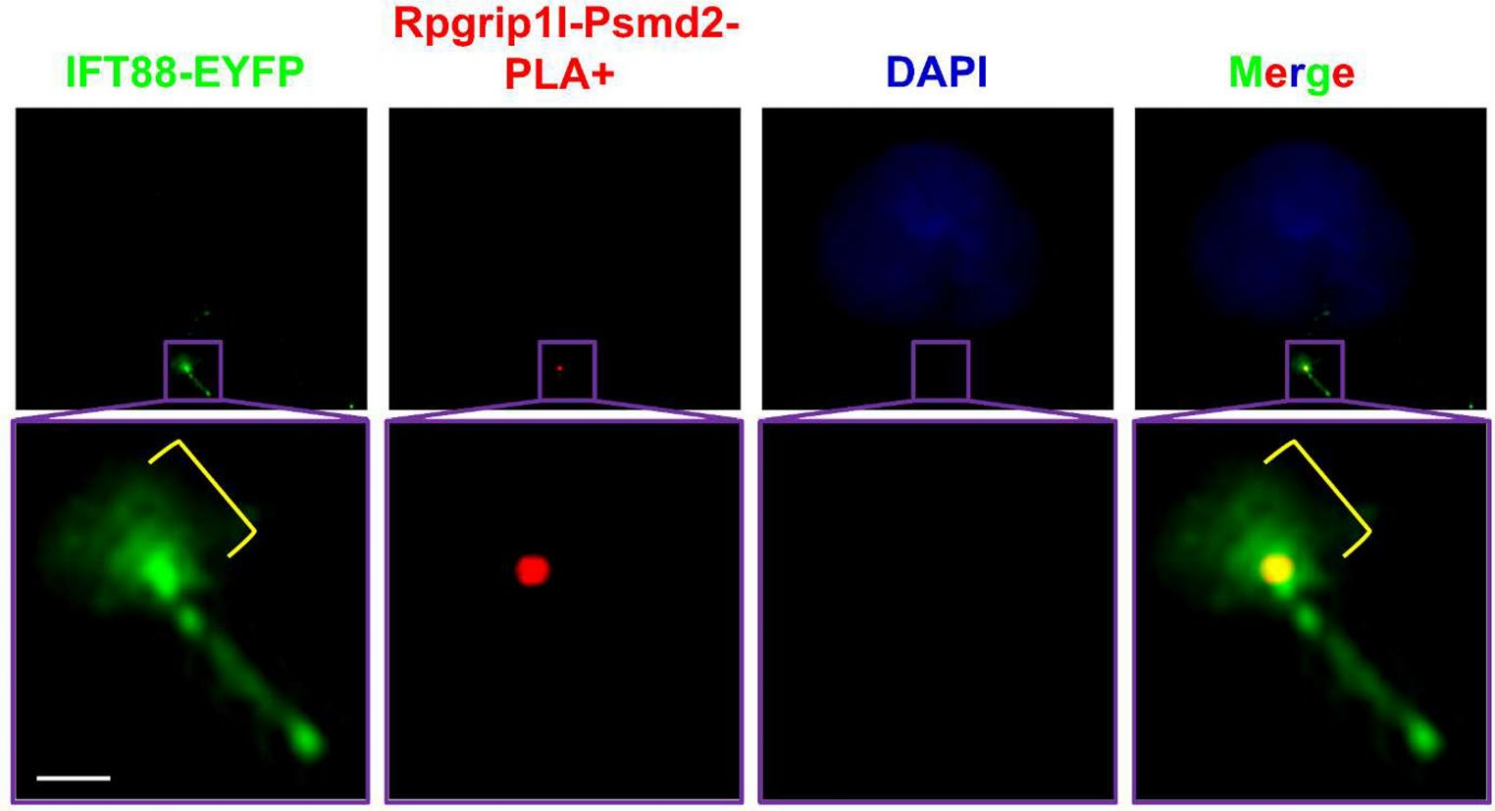
Exemplary result of an in situ PLA detecting close proximity of Rpgrip1l and Psmd2 at the ciliary base. In situ PLA on mouse embryonic fibroblasts isolated from wild-type embryos at E12.5. DAPI (blue) marks the cell nucleus, IFT88-EYFP (green) the ciliary base (indicated by the yellow bracket) and the axoneme. The red staining is a positive PLA signal demonstrating that Rpgrip1l and Psmd2 are in close proximity at the ciliary base. The scale bar (in white) represents a length of 1 μm

**Fig. 3:**
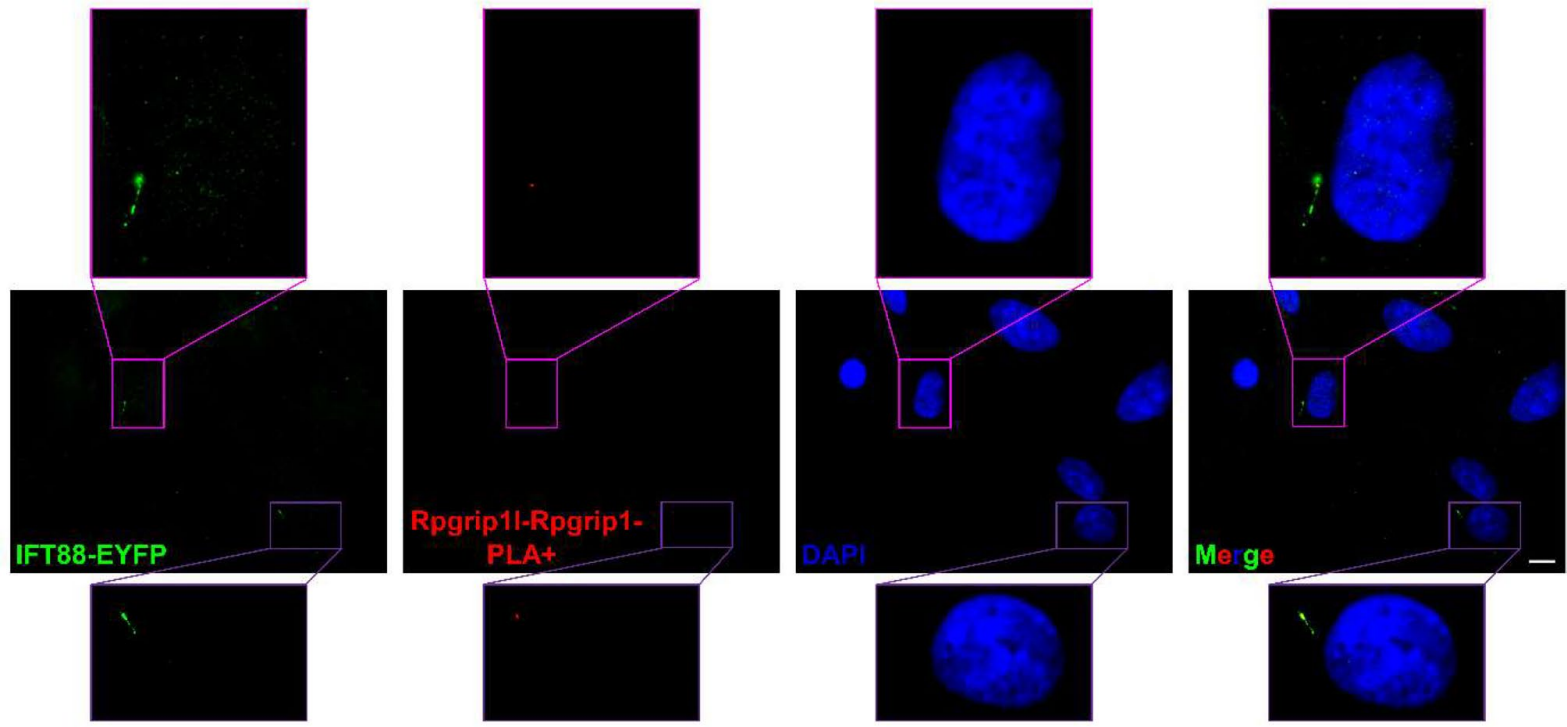
Exemplary result of an in situ PLA showing close proximity of Rpgrip1l and Rpgrip1 at the ciliary base. In situ PLA on mouse embryonic fibroblasts isolated from embryos at E12.5. DAPI (blue) marks the cell nucleus, IFT88-EYFP (green) the ciliary base and the axoneme. The red staining is a positive PLA signal demonstrating that Rpgrip1l and Rpgrip1 are in close proximity at the ciliary base. The scale bar (in white) represents a length of 10 μm. This is a modified version of an image published before [12]

**Fig. 4:**
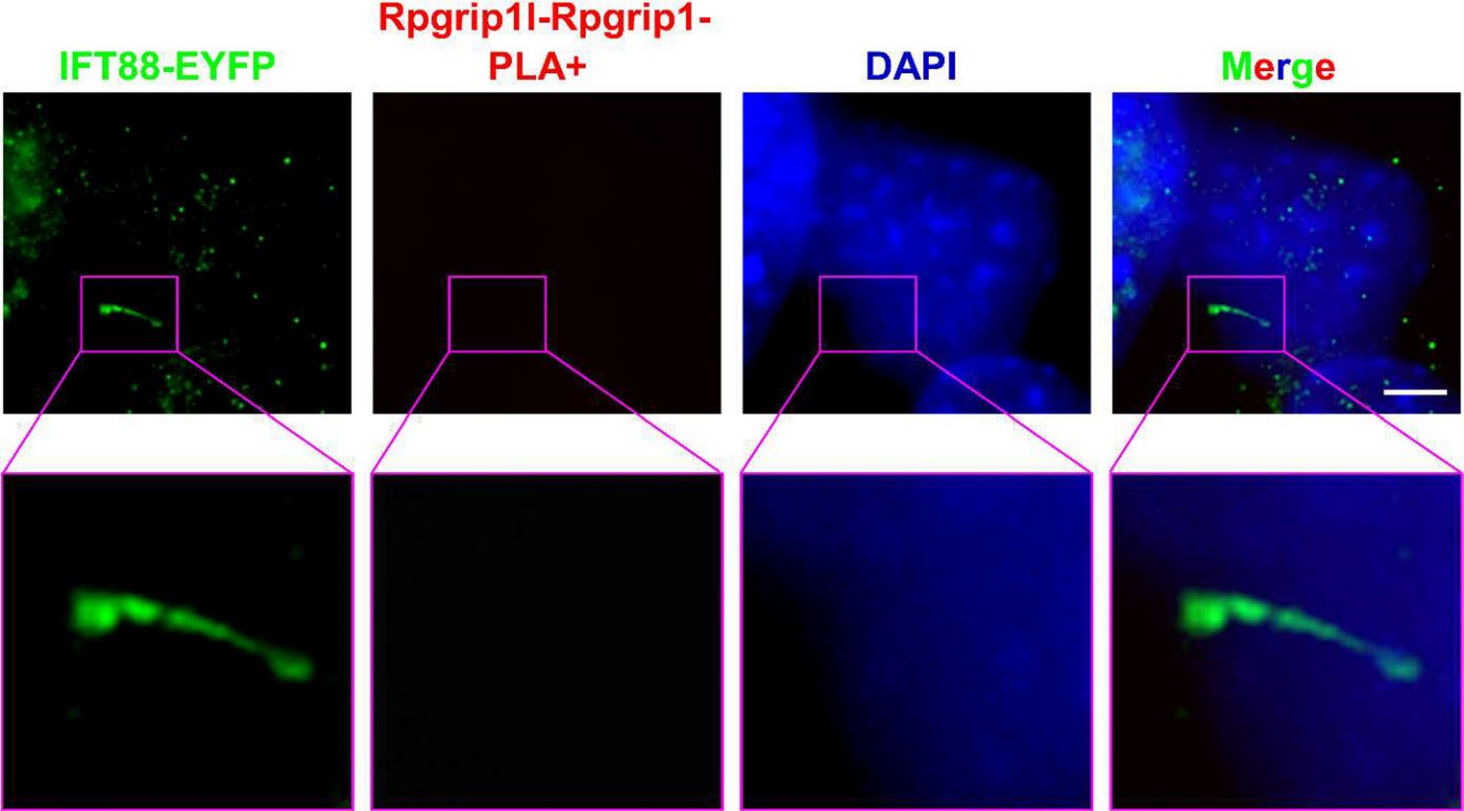
In situ PLA testing close proximity between Rpgrip1l and a truncated Rpgrip1 in primary cilia. In situ PLA on mouse embryonic fibroblasts isolated from *Rpgrip1* ^*nmf247/nmf247*^ embryos at E12.5. DAPI (blue) marks the cell nucleus, IFT88-EYFP (green) the ciliary base and the axoneme. No PLA signal is visible. In situ PLA was used to check whether Rpgrip1l and the truncated Rpgrip1 encoded by the *Rpgrip1* ^*nmf247*^ gene are localising in close proximity in primary cilia. The scale bar (in white) represents a length of 3 μm. This is a modified version of an image published before[12]

**Fig. 5:**
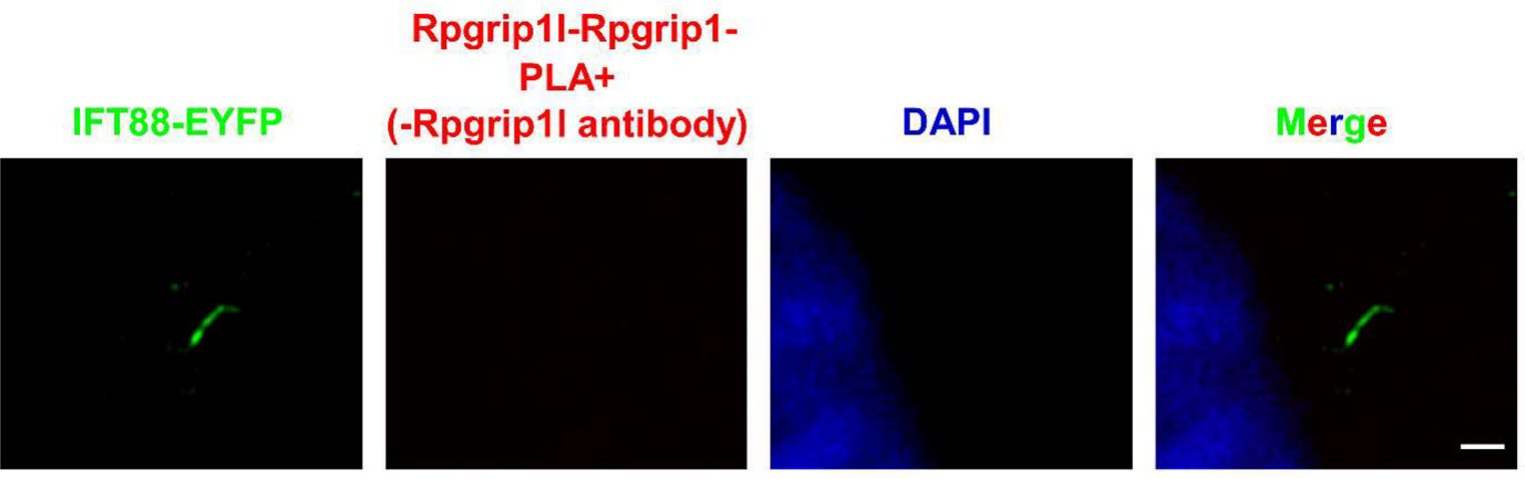
Reliability test of the in situ PLA technique. In situ PLA on mouse embryonic fibroblasts isolated from wild-type embryos at E12.5. DAPI (blue) marks the cell nucleus, IFT88-EYFP (green) the ciliary base and the axoneme. In situ PLA experiment was performed without using the antibody against Rpgrip1l but only with the antibody to Rpgrip1. No PLA signal was detected under these conditions. The scale bar (in white) represents a length of 1 μm

We used the in situ PLA technique to analyse whether a certain protein interaction takes place at primary cilia. By using in situ PLA, we were previously able to detect the well-known interaction between the ciliopathy proteins retinitis pigmentosa GTPase regulator-interacting protein 1‐like (Rpgrip1l) and nephrocystin 4 (Nphp4) in the transition zone [12]. Our former work led to the discovery of the ciliary proteasome whose activity is controlled by Rpgrip1l [13]. In search of the mechanism underlying the regulation of the ciliary proteasome by Rpgrip1l, we identified the proteasomal component Psmd2 as a novel interaction partner of Rpgrip1l by performing a yeast two-hybrid screen, co-immunoprecipitation and tandem affinity purification [13]. To investigate the subcellular localisation of the interaction between Rpgrip1l and Psmd2, we used in situ PLA. We have experienced that the application of in situ PLA does not allow an additional antibody staining using a primary antibody that is then recognised by a secondary antibody. The same experience was made by others [14]. In our in situ PLA studies, we marked cilia by transfecting a construct into MEFs which encodes a fusion protein of IFT88 and EYFP [12, 13]. As shown in Fig. 2, a distinct PLA signal (red signal) was detected at the base of the cilium, demonstrating that Rpgrip1l and Psmd2 are located in close proximity within the ciliary compartment and indicating that Rpgrip1l governs the activity of the ciliary proteasome by interacting with Psmd2 ^13^. By using the in situ PLA technique, we were also be able to reveal that the transition zone protein Rpgrip1l and the basal body protein retinitis pigmentosa GTPase regulator‐interacting protein 1 (Rpgrip1) localise close enough to interact with each other (Fig. 3). *Rpgrip1*^*nmf247/nmf247*^ mice produce a truncated Rpgrip1 protein [15]. In *Rpgrip1*^*nmf247/nmf247*^ MEFs, in situ PLA elucidated that Rpgrip1l and the truncated form of Rpgrip1 are not located in close proximity (Fig. 4). The reliability of the in situ PLA method is confirmed by the fact that no in situ PLA signal was detected when only the antibody against Rpgrip1 but not the antibody to Rpgrip1l is used in the in situ PLA experiment (Fig. 5).

## Discussion

The in situ proximity ligation assay (in situ PLA) represents a powerful technique to investigate protein-protein proximities within fixed cells and tissues with high spatial resolution. However, despite its robustness, in situ PLA is limited by several technical constraints and requires careful optimisation to achieve reliable and reproducible results.

One of the fundamental limitations of in situ PLA is that it cannot be applied in vivo, as it relies on antibody-based detection within fixed samples. Therefore, it is inherently restricted to endpoint analyses and cannot capture dynamic interactions in living systems. Further, a common challenge in in situ PLA experiments is the absence or weakness of the signal in otherwise positive samples. In such cases, the binding efficiency of the primary antibodies should be critically evaluated. Antibody incompatibility, low affinity, or incorrect dilutions may impair signal generation. In our experience, we always obtain a strong in situ PLA signal when we chose the dilution of the antibodies as if we were doing a conventional immunofluorescence staining. Additionally, suboptimal fixation, blocking, or buffer conditions can negatively influence antibody access and binding. Adjusting these parameters is often sufficient to restore signal strength. In our in situ PLA experiments, we achieved optimal in situ PLA results when we followed the fixation required by the respective antibody in a conventional immunofluorescence staining (in most cases, the cells were fixed in 4% paraformaldehyde for 1 h at 4 °C or in 100% methanol for 5 min at -20 °C). We used the buffer conditions according to the manufacturer’s instructions, since the in situ PLA experiments were reliable and successful under these conditions. In the Results section, we already mentioned that we were not able to perform a cilia-marking antibody staining in addition to the in situ PLA and thus we marked cilia by transfecting MEFs with a construct encoding a fusion protein of IFT88 and EYFP. However, Barbelanne and colleagues performed in situ PLA in RPE-1 cells with an additional antibody staining by using an anti-γ-tubulin-FITC antibody to mark centrosomes [16]. This antibody was added after washing twice with Wash Buffer B. Subsequently to the incubation in the anti-γ-tubulin-FITC antibody for 45 min, the RPE-1 cells were washed again with Wash Buffer B for 5 min. Future studies will show whether this obvious difference is based on cell type-specific conditions (MEFs vs. RPE-1 cells) or, more likely and promising, whether the coupling of a primary antibody with a fluorescent dye, such as FITC, opens up the possibility to label cilia without the need of expressing fluorescent fusion proteins. If these future studies confirm that a primary antibody coupled to a fluorescent dye provides a general and reliable option to mark subcellular structures in an in situ PLA experiment, it would facilitate the simultaneous labelling of subciliary structures (e.g. axoneme, basal body, transition zone). If it turns out that a primary antibody coupled to a fluorescent dye is not generally or reliably useful for this kind of labelling, subciliary structures could be simultaneously marked by co-expressing several fusion proteins, such as for example IFT88-EYFP for the labelling of the entire cilium and γ-tubulin-BFP for the labelling of the basal bodies and of centrosomes. Previously, we used an OFD1-EYFP fusion protein to mark basal bodies and centrosomes in an in situ PLA experiment [13]. To avoid false positive or false negative in situ PLA results, instrumental factors must also be considered. The use of incorrect filter sets on the fluorescence microscope can result in failure to detect the fluorescent signal. It is essential to ensure compatibility between the chosen fluorophore and the microscope’s excitation/emission filters. Furthermore, insufficient control of reaction conditions − particularly during the ligation and amplification steps − can affect PLA signal intensity. Deviations from the recommended incubation times or temperatures, as well as the use of inactive enzymes, can lead to false-negative results. Ligase and polymerase should be stored at -20 °C and freshly diluted before each use. Repeated freeze-thaw cycles or use of old dilutions should be avoided. Residual wash solution on the samples, especially before enzymatic steps, may dilute reagents or interfere with hybridisation and amplification, resulting in weak or absent signal. Complete removal of wash buffers using a suction apparatus is crucial before proceeding to subsequent steps. High background fluorescence is another frequent problem and may arise from various sources. One major cause is the use of an inappropriate or insufficient blocking solution. The chosen blocking reagent must be compatible with both the primary antibodies and PLA probes. We got excellent results by using 10% donkey serum in PBS containing 0.1% Triton X-100. Donkey serum was used as we optimised the protocol of the conventional immunofluorescence staining in the way that all our primary antibodies were generated in donkeys. Contact time between blocking agents and antibodies before application to the sample may also influence specificity. In paraffin-embedded samples, incomplete paraffin removal can lead to increased background, as can inadequate washing, particularly without gentle shaking. Moreover, allowing the samples to dry during the protocol can introduce artefacts and increase nonspecific staining. Moreover, non-specific antibody binding is a well-known cause of high background and can often be resolved by optimising fixation protocols, blocking strategies, or buffer compositions. Additionally, physical contaminants such as dust, salt crystals, or fixation precipitates can produce bright, non-specific fluorescent particles. Proper washing of cell cultures before fixation and maintenance of a clean working environment are necessary to minimise these artefacts. Another frequent artefact is a diffuse fluorescent mist over the entire sample. This may result from using ink-based pens to delineate tissue sections; instead, a grease pencil (e.g. ImmEdge™ Pen) should be used. Contaminated wash solutions or washing containers may also introduce background fluorescence and should be replaced immediately if contamination is suspected. An uneven signal distribution across the sample is sometimes observed and can be attributed to insufficient reagent coverage or reaction volume. Ensuring full coverage of the sample during each step and using appropriate volumes within the grease pencil boundary will promote uniform results. Finally, if nuclear staining is absent, the mounting medium may lack DAPI, or the fluorescence microscope may be configured with inappropriate filter settings. For DAPI, excitation at 360 nm and emission at 460 nm is required for optimal visualisation. In summary, while in situ PLA is a highly sensitive and specific technique for detecting protein proximities in situ, its success depends on strict adherence to protocol conditions, careful optimisation of antibody and buffer systems, and consistent attention to sample handling and reagent quality. Awareness of common pitfalls and systematic troubleshooting can significantly enhance data reliability and reproducibility.

The in situ PLA technique is a powerful tool ideally suited to analyse protein-protein interactions in a ciliopathy-relevant context. As mentioned above, many ciliopathies are caused by mutations in genes encoding transition zone proteins. Thus, the transition zone is an excellent example to illustrate the power of the in situ PLA method in investigating molecular causes of ciliopathies. In a previous work, we were able to demonstrate the close proximity of Rpgrip1l and Nphp4 in the vertebrate transition zone using in situ PLA. Moreover, our work revealed that a complete loss of Rpgrip1l results in a severly reduced amount of Nphp4 in the transition zone showing the role of Rpgrip1l in recruiting Nphp4 to the transition zone [12]. However, many ciliopathies are caused by a truncation of a ciliary protein rather than by a complete loss of this protein. Rpgrip1l localises to the transition zone by its N-terminal CC domains and interacts with Nphp4 via its C-terminal C2 domain. If the CC domains and/or this C2 domain are truncated, the question arises whether the interaction of Rpgrip1l and Nphp4 in the transition zone is still existing. While methods, such as co-immunoprecipitation and tandem affinity purification, are only able to ascertain whether the interaction between the truncated Rpgrip1l and Nphp4 still functions, the in situ PLA techniques allows a statement about the spatial kind of this interaction. For instance, co-immunoprecipitation might reveal that the truncated Rpgrip1l and Nphp4 still interact with each other, while in situ PLA indicates that this interaction occurs outside the transition zone, elsewhere in the cell. In this way, the in situ PLA technique can offer valuable insights into the molecular mechanisms underlying ciliopathies.

## Conclusions

In situ PLA is a valuable and sensitive method for detecting protein-protein interactions with spatial resolution in fixed cells and tissues. It is particularly suited for studying molecular complexes in subcellular compartments such as the ciliary transition zone. While the technique offers high specificity and fluorescence-based visualisation of protein proximities, its reliability strongly depends on optimised experimental conditions and rigorous protocol adherence. Factors such as antibody quality, enzyme activity, incubation parameters, and appropriate imaging settings are critical for signal quality. By identifying and addressing potential sources of error, researchers can fully exploit the potential of in situ PLA to explore complex protein networks within cellular structures. As a result, in situ PLA serves as a robust tool to complement biochemical interaction studies and enhance our understanding of spatial protein organisation in health and disease.

## Data Availability

No datasets were generated or analysed during the current study.
